# Comparison of Treatment Outcomes of Selective Laser Trabeculoplasty for Primary Open-Angle Glaucoma and Pseudophakic Primary Angle-Closure Glaucoma Receiving Maximal Medical Therapy

**DOI:** 10.3390/jcm10132853

**Published:** 2021-06-28

**Authors:** Pei-Yao Chang, Jiun-Yi Wang, Jia-Kang Wang, Tzu-Lun Huang, Yung-Ray Hsu

**Affiliations:** 1 Department of Ophthalmology, Far Eastern Memorial Hospital, Ban-Chiao, New Taipei City 220, Taiwan; jiakangw2158@gmail.com (J.-K.W.); huang.tzulum@gmail.com (T.-L.H.); scherzoray@gmail.com (Y.-R.H.); 2 Department of Medicine, National Taiwan University Hospital, Taipei City 100, Taiwan; 3 Department of Healthcare Administration, Asia University, Taichung City 413, Taiwan; jjwang@asia.edu.tw; 4 Department of Electrical Engineering, Yuan Ze University, Taoyuan City 320, Taiwan; 5 Department of Medical Research, China Medical University Hospital, China Medical University, Taichung City 404, Taiwan

**Keywords:** selective laser trabeculoplasty, intraocular pressure, glaucoma

## Abstract

Selective laser trabeculoplasty (SLT) is a useful treatment for intraocular pressure (IOP) control. However, there are only a few reports which compare the outcomes of SLT between primary open-angle glaucoma (POAG) and primary angle-closure glaucoma (PACG). We compared the efficacy of SLT for patients with PACG following phacoemulsification with POAG receiving maximal medical therapy (MMT). Consecutive glaucoma patients followed up for at least 1 year after SLT were retrospectively evaluated and IOP reductions at 6 months and 12 months were analyzed. Seventy-six patients were included in the analyses. The baseline IOPs in the POAG and PACG group were 18.5 ± 3.3 mmHg and 16.9 ± 2.5 mmHg, respectively, with 2.8 ± 0.9 and 2.7 ± 0.8 types of IOP lowering medication. The average IOP at the 6-month and 12-month follow-up after SLT was significantly decreased and comparable in both the POAG and PACG groups. For those with a low baseline IOP, the effect of SLT on IOP reduction at 12 months was significantly better in the PACG than in the POAG group (*p* = 0.003). IOP reduction at 6 and 12 months after SLT was significantly greater in those with a high baseline IOP than those with a low baseline IOP (*p* < 0.0065). In summary, the one-year efficacy of SLT was equivalent in POAG and pseudophakic PACG patients receiving MMT; however, SLT was more effective in eyes with PACG than eyes with POAG when focusing on those with a lower baseline IOP.

## 1. Introduction

Intraocular pressure (IOP) control is currently the only well-established treatment for glaucoma to slow or prevent further visual field progression. Selective laser trabeculoplasty (SLT) is an efficient IOP-lowering treatment for primary open angle glaucoma (POAG) and ocular hypertension [1,2]. Studies have proved that SLT can be used as an initial treatment or in combination with IOP-lowing drugs when the topical medications could not obtain satisfactory therapeutic effects in patients with POAG [1,3,4,5].

Primary angle closure glaucoma (PACG) is common in Asia. Laser peripheral iridotomy (LPI) is the current first-line treatment in the management of PACG and argon laser peripheral iridoplasty has also been shown to dramatically lower the IOP and open up the closed angles [6]. However, medical therapy is often required after LPI for IOP control [7]. Studies have reported that SLT is a safe and cost-effective modality for reducing IOP in post LPI PACG [8,9,10]. A multicenter randomized trial demonstrated the comparable effect in PACG patients between SLT treatment and topical prostaglandin analog usage [8]. Therefore, SLT is also a potential therapeutic option in patients with PACG.

Cataract and glaucoma frequently coexist in elderly PACG patients. Numerous studies have demonstrated a modest reduction in IOP and angle widening following cataract surgery in patients with PACG [11,12,13]. However, trabecular damage may result in IOP elevation even after opening of the anterior chamber angles by cataract surgery in eyes with long-standing iridotrabecular apposition [14]. Patients with severe glaucomatous optic neuropathy and IOP fluctuation even following cataract surgery may require additional IOP-lowering treatment, including long-term glaucoma medications or even surgical intervention. Tham et al. reported both phacoemulsification and trabeculectomy were effective in reducing IOP in chronic angle closure glaucoma, but 19% of patients from the phacoemulsification arm did eventually require trabeculectomy in the 2-year follow-up period [15]. SLT has fewer complications, as compared to surgery, whether trabeculectomy or even minimally invasive glaucoma surgery [8,15]. SLT addresses the issue of compliance associated with topical medications and offers a treatment option in a select group of patients with PACG whose angle has been widened after lens removal but still having unsatisfactory IOP control.

Limited studies have been evaluated regarding SLT as an adjunctive treatment modality for PACG. Therefore, we performed a retrospective study in a mid-term follow-up of one year to observe the outcomes of SLT in patients with PACG following phacoemulsification and intraocular lens implantation (PEA + IOL) and compared the outcomes of SLT in patients with POAG.

## 2. Materials and Methods

This was a retrospective chart review study, which included patients who had SLT performed by one glaucoma specialist (PYC) between January 2015 and December 2018. The study protocol was approved by the Ethics Committee of our institution (No.: 109108-E) and the study adhered to the tenets of the Declaration of Helsinki. Baseline demographic data such as age, gender, IOP, visual field mean deviation, total laser power, types of glaucoma, history of ocular surgery, and the number of glaucoma medications were collected from medical charts.

The IOP usually significantly decreases in angle closure glaucoma patients following cataract surgery [11,12,13]. Most PACG patients choose conservative treatments (topical medications) following PEA+IOL to control IOP, and SLT was the most considered treatment option when the maximal medical therapy (MMT) failed in these circumstances in our hospital. Moreover, we performed SLT mostly as an adjunct treatment with unsatisfactory IOP control with visual field progression in patients with POAG. Therefore, the current study included patients with POAG or PACG with unsatisfactory IOP control with visual field progression even receiving MMT or patients without visual field progression but intolerability to MMT. The degree of SLT treatment was variable in PACG patients, depending on extent of remaining peripheral anterior synechia (PAS) following phacoemulsification. Because we routinely performed 270° SLT treatment in open angle glaucoma, we included PACG patients only when they had at least 270° of trabecular meshwork (TM) visible by gonioscopy without corneal indentation or manipulation after uncomplicated phacoemulsification.

Exclusion criteria were secondary glaucoma, previous ophthalmic surgery, except for uncomplicated cataract surgery, and previous ophthalmic laser, except LPI. However, LPI and uncomplicated phacoemulsification must be performed at least 12 months prior to SLT treatment. We excluded any other form of open angle glaucoma other than POAG, such as pigmentary glaucoma or pseudoexfoliation glaucoma. Filtering surgery instead of SLT was usually considered in such cases in our routine practice.

SLT was performed using the Selecta Duet laser (Lumenis, Dreieich, Germany) (frequency doubled, Q-switched Nd: YAG 532 nm, 3-ns pulse, spot size 400 μm). The initial power setting was between 0.6 and 0.8 mJ and the energy was increased or decreased by 0.1 mJ until a small bubble formation appeared for the remaining treatment. The procedure was then completed for 270°, avoiding areas of PAS and areas with limited visibility of the TM. A drop of brimonidine 0.2% was administered after laser therapy. IOP was measured 1 h after the procedure and patients who experienced a postoperative elevation of >5 mm Hg received oral carbonic anhydrase inhibitors.

Patients did not receive any topical anti-inflammatory medications and maintained the same preoperative IOP lowering drugs before the next visit. The amount of glaucoma medications prescribed in the postoperative period was decided according to the IOP level and glaucoma severity. Postoperative IOP data were collected at the 3-, 6-, 9- and 12-month visits with the noncontact computerized tonometer (CT-80, Topcon, Tokyo, Japan).

### Statistical Analysis

Statistical analysis was performed using the SPSS software (v.22.0; IBM Corp, Armonk, NY, USA) Descriptive statistics such as the number and percentage for categorical data and the mean ± standard deviation for continuous data were used to present data distributions. Chi-squared test and the two-sample t test were respectively used to compare sample proportions and sample means of the two groups of POAG and PACG. The amount of ocular hypotensive drugs used before and after SLT was compared. IOP reductions from the baseline to the follow-up time-points were calculated and tested, based on paired *t*-test. Nonparametric methods such as Mann−Whitney U test and Wilcoxon signed-rank test were also conducted for a double check, due to the small to moderate sample size in the study. Considering that there were 5 and 9 missing IOP in the POAG group and 2 and 3 missing IOP in the PACG group for 6- and 12-month follow-up, respectively, the generalized estimating equations (GEE) analysis was implemented for repeated measures. It was used to evaluate whether the IOP reduction effect (at the time-points of 6 and 12 months) was different between groups, either open and closed type, high baseline IOP (IOP ≥ 17 mmHg) and low baseline IOP (IOP < 17 mmHg) group, or better VF (MD)and worse VF group. In each GEE model, age, sex, baseline IOP, and VF were included as control variables when appropriate, and the interaction terms of time and group were also included. A significance of an interaction in the model implies that the effect was different between groups. The significance level of all analyses was set at 0.05, except for comparisons of IOP changes between POAG and PACG patients for overall and subgroups, which was set at 0.0065 (=0.05/8) due to Bonferroni correction for multiple testing.

## 3. Results

### 3.1. Demographics of the Participants

Baseline characteristics, clinical parameters and topical medications of the study participants were displayed in Table 1. There were 53 participants with POAG and 23 with PACG included in the analyses. The mean age (51.7 ± 10.9 vs. 66.3 ± 10.2 years old) and percentages of male (32.1% vs. 60.9%) were both significantly different between the groups of POAG and PACG. Significantly worse VF, lower baseline IOP and higher presence of PAS in the PACG group were observed. However, there was no significant difference in the indications for SLT between the two groups. Table 2 shows that the number of bottles and the types of ocular hypotensive drugs, as well as the proportion of each of the four types of drugs, had no significant difference after SLT in either group.

### 3.2. IOP Change

The IOP change is displayed in Table 3. The average IOP at the 6-month and 12-month follow-up after SLT had significantly decreased in both POAG and PACG group. When we further divided patients into a high baseline IOP (IOP ≥ 17 mmHg) and a low baseline IOP (IOP < 17 mmHg) group, the average IOP decreased significantly for those with a high baseline IOP in both the POAG and PACG group. For those with a low baseline IOP, it had decreased significantly at the 12-month follow-up after SLT only in the PACG group.

Table 4 summarizes the results of the interaction terms from the GEE analyses. It demonstrated that the interactions of time and the types of glaucoma were not significant (*p* = 0.327 and 0.135 at 6 and 12 months). For those with low baseline IOP, the effects on IOP reduction at 12 months after SLT were significantly better in the PACG than in the POAG group (*p* = 0.003). In patients with POAG, the IOP reduction at both 6 and 12 months after SLT was significantly more in the high baseline IOP group than the low baseline IOP group, while the effects on reducing IOP at both 6 and 12 months after SLT had no significant difference between the better and worse VF group.

None of the eyes experienced IOP elevation of >10 mm Hg, whereas 2 (3.8%) eyes with POAG had 5 mm Hg IOP elevation within 1 h of SLT. No permanent adverse effects of SLT were noted in any of the patients.

## 4. Discussion

Studies comparing the effectiveness of SLT in PACG and POAG are inconclusive and the data regarding the efficacy in PACG eyes is rather limited. We have summarized the efficacy of SLT in patients with PACG in Table 5. Most studies reporting the efficacy of SLT in PACG patients did not focus on PACG patients following cataract surgery [8,9,10,16]; while this is the first study which evaluated PACG patients only following PEA+IOL. Natalia IK [16] reported there was no significant difference in the one-year efficacy of SLT between POAG and PACG, which was similar to our findings when we did not divide patients into high and low IOP group. However, they excluded patients who underwent phacoemulsification and their baseline IOP prior to SLT was 23.18 ± 3.53 mmHg in PACG and 22.23 ± 2.99 mmHg in POAG, which was higher than that in our study population. Ali Aljasim et al. [8] showed that the success rate of SLT in PACG was better than that in POAG, although it did not reach a significant level. One thing in their study that needs to be addressed was that the percentage of 360-degree of SLT application and the laser energy was significantly higher in their POAG group, while the success rate was 84% in the PACG and 79% in the POAG group. Our study highlighted SLT had better efficacy in patients with PACG compared to POAG, but was only found in patients with IOP less than 17 mmHg. We found in the group of PACG with IOP less than 17 mmHg, IOP was 14.5 ± 1.0 mmHg at baseline and 12.0 ± 1.6 mmHg 1 year after SLT treatment, the IOP reduction percentage (13.3%) in the PACG group was significantly more than that in POAG group (3.2%).

The injuries to the conventional aqueous outflow system induced by angle closure are variable and depend on various anatomic factors [6]. The mechanisms of angle closure can be divided into three groups: (i) appositional, leading to pretrabecular obstruction without TM injury; (ii) appositional with TM obstruction associated with structural and functional modifications due to chronic contact; (iii) synechial, accompanied by permanent adherence [18]. We excluded angle closure patients who had a history of acute attack and included those only with visible TM over 270°. It is impossible to differentiate type (i) and (ii) by clinical examinations, but patients in the group of PACG with IOP less than 17 mmHg after PEA + IOL would probably be those who had less TM injury induced by angle closure. Moreover, TM height was reported to be shorter in PACG patients compared to POAG patients [19]. Since the spot size and duration of SLT was fixed, the laser energy might be more concentrated in a smaller tissue. As for the reason why SLT was more effective in the low IOP group in PACG only at the 1-year follow-up but not at the 6-month follow-up was unclear. However, it was reported that Raj et al. [10] found the percentage of eyes without medication achieving IOP in the level of 12–15 mmHg was 9% at 6 months but increased to 35.7% at the 1-year follow-up in PACG patients.

The published literature reporting the outcomes of SLT varies widely with IOP reductions ranging from 6.9% up to 35.9% of baseline IOP [1,2,3,4,5,8,9,10,20,21,22,23,24]. Primary SLT achieved IOP reductions of 29.7% in OHT eyes and 26.1% in OAG eyes [1]. IOP reduction varied widely depending on different study populations, baseline IOP and the degree of laser application. The IOP reduction percentage in the POAG group was relatively low in our study, which was 15.5% at the 6-month and 13% at the 12-month follow-up. Our baseline IOPs (18.5 ± 3.3 mmHg in POAG group and 16.9 ± 2.5 mmHg in PACG group) were lower compared with those in previous studies. Most of the evidence pointed at a higher success rate and/or greater IOP reduction in eyes with a higher baseline IOP [3,16,19,21], and we also had the same findings in both groups, although it only reached the significant level in the POAG group. SLT achieved more than 20% IOP reduction in 95% of eyes in medically controlled glaucoma patients with a 1.5 baseline number of medications [4]. However, greater usage of hypotensive eye drops before SLT was associated with a higher risk of failure for both POAG and PACG patients [16]. In our study, patients used MMT with approximately 2.1 bottles/2.7 types of hypotensive eye drops before SLT treatment. The one-year efficacy of SLT was very limited in patients under MMT with only 14.2% of patients reaching IOP reduction > 20% [5], which was in agreement with our findings. The amount of ocular hypotensive drugs before and after SLT was not significantly different in this study because we performed SLT mainly as an adjunctive therapy. We did not reduce the current medications despite successful SLT since most of the patients in this study had moderate to severe glaucoma.

In some respects, the baseline characteristics were not similar between the two groups. All of the PACG patients while only 7.5% of the POAG patients were pseudophakic in our cohort. However, the IOP-lowering effect of SLT treatment was reported to be comparable between pseudophakic and phakic eyes in prospective [19] and retrospective studies [23,24]. Moreover, the baseline VF defect was significantly more and the baseline IOP was significantly less in our PACG group than that in our POAG group, which was quite reasonable that lower target IOP was usually set in severer patients. In our hospital, we were more conservative when considering SLT as a supplementary treatment in PACG patients who had received cataract surgery based on the following two reasons: first, good outcomes in terms of IOP control have been found following lens extraction for PACG [10]. Numerous studies even suggest PEA+IOL is a feasible option for IOP control in PACG [13]. Second, slower VF progression rate in PACG compared to high tension glaucoma and normal tension glaucoma when baseline severity was matched [25]. Therefore, if the IOP and VF were stable after PEA+IOL, SLT would not be suggested unless topical antiglaucoma medication was not tolerable.

Our study has its limitations including retrospective design, lack of information of angle pigmentation, a small sample size and a limited follow-up period. Moreover, the baseline demographics were different between the two groups. However, we adjusted gender, age and even baseline IOP in relevant analyses, and we included only those PACG patients with at least 270° of TM visible by gonioscopy, therefore the extent of laser application was the same in the two groups. Lastly, Goldman applanation tonometer was the gold standard of IOP measurement, but mostly we used it in prospective glaucoma studies. Due to the retrospective design, a noncontact tonometer was used in this study. In summary, the one-year efficacy of SLT was equivalent in POAG and PACG patients receiving MMT; however, SLT was more effective in eyes with PACG than eyes with POAG when focusing on those with a lower baseline IOP. Although our sample size is fairly small to reach definitive conclusions, it is worthy of note that these groups of patients may still benefit from SLT. Our results suggest clinicians may consider SLT as a supplementary treatment in eyes with PACG after PEA+IOL even when the IOP is lower than 17 mmHg. Further studies would be required to explore the long-term effect of SLT in PACG patients with low IOP following cataract surgery.

## 5. Conclusions

In summary, the one-year efficacy of SLT was equivalent in POAG and PACG patients receiving MMT; however, SLT was more effective in eyes with PACG than eyes with POAG when focusing on those with a lower baseline IOP.

## Figures and Tables

**Table 1:** Baseline characteristics of SLT treated eyes in PACG and POAG groups.

	POAG	PACG	*p*-Value ^a^
Number of patients	53	23	
Age (years)	51.7 ± 10.9	66.3 ± 10.2	<0.001
Male gender	17 (32.1%)	14 (60.9%)	0.024
Mean deviation (dB)	−11.3 ± 7.3	−16.3 ± 7.7	0.011
IOP (mmHg)	18.5 ± 3.3	16.9 ± 2.5	0.026
Central corneal thickness (μm)	565.9 ± 31.1	555.6 ± 33.6	0.224
Circumpapillary retinal nerve fiber layer (μm)	69.3 ± 12.7	65.0 ± 9.6	0.129
Pseudophakic	4 (7.5%)	23 (100%)	<0.001
Presence of peripheral anterior synechia	0 (0%)	4 (17.4%)	0.007
Indication for SLT			1.000
Uncontrolled IOP	45 (84.9%)	20(87.0%)	
Intolerability to Topical medication	8 (15.1%)	3 (13.0%)	
Topical medication			
Bottle	2.1 ± 0.6	2.1 ± 0.5	0.851
Type	2.8 ± 0.9	2.7 ± 0.8	0.530
A-agonist	24 (45.3%)	15 (65.2%)	0.138
β-blocker	45 (84.9%)	16 (69.6%)	0.208
Prostaglandin analog	47 (88.7%)	20 (87.0%)	1.000
Carbonic anhydrase inhibitor	34 (64.2%)	11 (47.8%)	0.211

**^a^***p*-values of two-sample t test. The results of testing for continuous data based on Mann−Whitney U test were the same and thus not shown here.

**Table 2:** Comparisons of the amount of ocular hypotensive drugs before and one year after SLT.

	Before SLT		After SLT		
	Mean ± SD	(%)	Mean ± SD	(%)	*p*-Value ^a^
**POAG**					
Bottle	2.1 ± 0.6		2.2 ± 0.6		0.419
Type	2.8 ± 0.9		2.9 ± 0.9		0.444
A-agonist		45.3%		45.3%	1.000
β-blocker		84.9%		84.9%	1.000
Prostaglandin analog		88.7%		90.6%	1.000
Carbonic anhydrase inhibitor		64.2%		67.9%	0.727
**PACG**					
Bottle	2.1 ± 0.5		2.1 ± 0.6		1.000
Type	2.7 ± 0.8		2.7 ± 1.0		0.665
A-agonist		65.2%		69.6%	0.629
β-blocker		69.6%		73.9%	1.000
Prostaglandin analog		87.0%		95.7%	0.500
Carbonic anhydrase inhibitor		47.8%		34.8%	0.250

**^a^***p*-values of paired t test. The results of testing for continuous data based on Wilcoxon signed-rank test were the same and thus not shown here.

**Table 3:** IOP values and the reduction before and after SLT of all cases and subgroups of higher and lower IOP. (High IOP: baseline IOP ≥ 17 mmHg; low IOP: baseline IOP < 17 mmHg.).

Group Time-Point	*n*	IOP (Mean ± SD)	IOP Reduction (Mean ± SD)	IOP Reduction Percentage (%)	*p*-Value ^a^
POAG (all cases)					
Baseline	53	18.5 ± 3.3			
6 months	48	15.6 ± 3.2	2.9 ± 3.4	15.5%	<0.001
12 months	44	16.1 ± 3.5	2.7 ± 3.1	13.0%	<0.001
POAG (high IOP)					
Baseline	36	20.2 ± 2.6			
6 months	32	16.3 ± 3.0	3.9 ± 3.2	19.1%	<0.001
12 months	31	16.7 ± 3.7	3.6 ± 3.1	16.9%	<0.001
POAG (low IOP)					
Baseline	17	15.0 ± 1.1			
6 months	16	14.2 ± 3.2	0.8 ± 2.7	4.9%	0.250
12 months	13	14.5 ± 2.3	0.5 ± 1.6	3.2%	0.261
PACG (all cases)					
Baseline	23	16.9 ± 2.5			
6 months	21	14.8 ± 2.7	2.1 ± 3.1	12.6%	0.005
12 months	20	13.3 ± 2.2	3.3 ± 1.9	21.5%	<0.001
PACG (high IOP)					
Baseline	14	18.5 ± 1.9			
6 months	12	15.4 ± 2.6	3.3 ± 2.9	16.5%	0.002
12 months	11	14.3 ± 2.1	4.1 ± 2.0	22.6%	<0.001
PACG (low IOP)					
Baseline	9	14.5 ± 1.0			
6 months	9	13.9 ± 2.7	0.6 ± 2.7	3.1%	0.545
12 months	9	12.0 ± 1.6	2.5 ± 1.5	13.3%	<0.001

**^a^***p*-values of paired t test. The results of testing based on Wilcoxon signed-rank test were the same and thus not shown.

**Table 4:** Comparisons of IOP changes between POAG and PACG patients for overall and subgroups (A) and between patients with high and low VF or higher and lower baseline IOP for subgroups of POAG and PACG (B), based on the GEE analysis. All models included the main effects of the interaction terms and were adjusted for age, sex, pre IOP group, and VF group where appropriate.

Model		95%CI		
Subgroup Interaction Term	B	Lower Limit	Upper Limit	*p*-Value
(A)				
All patients				
(PACG vs. POAG) and (6th month vs. baseline)	0.78	−0.78	2.35	0.327
(PACG vs. POAG) and (12th month vs. baseline)	−0.98	−2.26	0.30	0.135
1. Baseline IOP ≥ 17 mmHg				
(PACG vs. POAG) and (6th month vs. baseline)	0.71	−1.17	2.59	0.459
(PACG vs. POAG) and (12th month vs. baseline)	−0.78	−2.53	0.97	0.381
2. Baseline IOP < 17 mmHg				
(PACG vs. POAG) and (6th month vs. baseline)	0.23	−1.87	2.33	0.828
(PACG vs. POAG) and (12th month vs. baseline)	−1.85	−3.07	−0.63	0.003
3. Visual field defect ≥ 12 dB				
(PACG vs. POAG) and (6th month vs. baseline)	0.76	−1.42	2.94	0.495
(PACG vs. POAG) and (12th month vs. baseline)	−1.31	−3.00	0.39	0.130
4. Visual field defect < 12 dB				
(PACG vs. POAG) and (6th month vs. baseline)	0.46	−1.49	2.41	0.644
(PACG vs. POAG) and (12th month vs. baseline)	−0.68	−2.68	1.33	0.509
(B)				
1. POAG				
(VF: higher vs. lower) and (6th month vs. baseline)	0.16	−1.73	2.06	0.867
(VF: higher vs. lower) and (12th month vs. baseline)	0.54	−1.21	2.29	0.545
2. PACG				
(VF: higher vs. lower) and (6th month vs. baseline)	0.41	−1.91	2.73	0.730
(VF: higher vs. lower) and (12th month vs. baseline)	−0.16	−1.89	1.56	0.852
3. POAG				
(pre IOP: higher vs. lower) and (6th month vs. baseline)	−3.03	−4.72	−1.35	<0.001
(pre IOP: higher vs. lower) and (12th month vs. baseline)	−2.94	−4.24	−1.63	<0.001
4. PACG				
(pre IOP: higher vs. lower) and (6th month vs. baseline)	−2.49	−4.78	−0.20	0.033
(pre IOP: higher vs. lower) and (12th month vs. baseline)	−1.71	−3.32	−0.10	0.038

**Table 5:** Summary of efficacy of SLT in patients with primary angle-closure glaucoma.

Paper	Design	Number of Eyes	Postoperative Follow-up	Definition of Success	Success Rate	Average IOP Reduction
Ali Aljasim et al. [8] (2016)	Retrospective case–control study	*n* = 59 (PAC/PACG), *n* = 59 (POAG)	PAC/PACG: 6–20 months POAG: 6–17 months	IOP reduction ≧ 20% without further medical or surgical intervention or a reduction in the number of glaucoma medications by ≧1 while maintaining the target IOP	PAC/PACG: 84.7%, POAG: 79.6% *p* = 0.47	IOP reduction in patients with uncontrolled IOP: 38% (PAC/PACG) vs. 32.7% (POAG), *p* = 0.08
Narayanaswamy et al. [9] (2015)	Randomized clinical trial	*n* = 50 (SLT), *n* = 50 (PGA)	6 months	Complete success: IOP lower than 21 mmHg without any additional IOP-lowering medications Qualified success: IOP lower than 21 mmHg who required IOP lowering medication	Complete success: 60% (SLT) vs. 84% (PGA), *p* = 0.008 Qualified success: 18% (SLT) vs. 6% (PGA), *p* = 0.06	16.9% (SLT) vs. 18.5% (PGA) *p* = 0.52
Raj et al. [10] (2018)	Prospective cross-sectional study	*n* = 34 (23 PAC and 11 PACG)	1 year	N/A	N/A	3 month: 19.61% 6 month: 22.43% 1 year: 28.7%
Kurysheva et al. [16] (2018)	Retrospective case–control study	*n* = 68 (PACG), *n* = 74 (POAG)	PACG/PACG: 6.94 ± 1.92 years POAG: 6.34 ± 1.94 years	20% IOP reduction with topical hypotensive medications without any hypotensive intervention (repeated SLT, antiglaucoma surgery, phacoemulsification of cataracts)	PACG vs. POAG 2 years: 66% vs. 62% 3 years: 62% vs. 54% 4 years: 44% vs. 38% 5 years: 42% vs. 36% 6 years: 6% vs. 4% *p* = 0.24	At 6 years, reduction in mean baseline IOP from 23.57 ± 2.30 to 18.77 ± 2.25 (PACG) and from 22.45 ± 1.46 to 18.86 ± 2.09 (POAG)
Kurysheva et al. [17] (2019)	Prospective longitudinal study	*n* = 60 (PACG), *n* = 64 (POAG)	PACG: 6.75 ± 1.83 years POAG: 6.22 ± 1.54 years	20% IOP reduction with topical hypotensive medications without any hypotensive intervention (repeated SLT, antiglaucoma surgery, and phacoemulsification).	PACG vs. POAG 1 year: 89% vs. 90% 6 year: 34% vs. 36%	N/A ^a^

^a^ N/A: not applicable (IOP reduction percentage was not shown in this study).

## Data Availability

Data will not be shared because the authors are performing other analyses that have not yet been published.
